# Heart valve service provision in the United Kingdom and the effect of the COVID 19 pandemic; improved but must do better. A British Heart Valve Society national survey

**DOI:** 10.1186/s44156-024-00047-y

**Published:** 2024-05-07

**Authors:** R. Mohindra, L. E. Dobson, D. Schlosshan, P. Khan, B. Campbell, M. Garbi, B. Chambers, J. B. Chambers

**Affiliations:** 1 Blackpool Victoria Hospital, Blackpool, UK; 2 Manchester University Foundation Trust, Manchester, UK; 3 Leeds Teaching Hospitals NHS Trust, Leeds, UK; 4 British Heart Valve Society, London, UK; 5 Guys and St Thomas’ NHS Foundation Trust, London, UK; 6 Royal Papworth Hospital, Cambridge, UK

**Keywords:** Valuvular Heart Disease, Endocarditis, Cardiovascular Disease, Cardiology, Imaging, Echocardiology

## Abstract

**Background:**

Outpatient care for patients with heart valve disease (HVD) is best provided by valve clinics delivered by specialists. Modern day practice in the United Kingdom (UK) is currently poorly understood and has not been evaluated for nearly a decade. Furthermore, the COVID 19 pandemic changed the management of many chronic diseases, and how this has impacted patients with heart valve disease is unclear.

**Methods:**

A British Heart Valve Society survey was sent to 161 hospitals throughout the UK.

**Results:**

There was a general valve clinic in 46 of the 68 hospitals (68%), in 19 of 23 Heart Centres (83%) and 29 of 45 DGHs (64%). Across all settings, 3824 new patients and 17,980 follow up patients were seen in valve clinics per annum. The mean number of patients per hospital were 197 (median 150, range 48–550) for new patients and 532 (median 400, range 150–2000) for follow up. On the day echocardiography was available in 55% of valve clinics. In patients with severe HVD, serum brain natriuretic peptide (BNP) was measured routinely in 39% of clinics and exercise testing routinely performed in 49% of clinics. A patient helpline was available in 27% of clinics. 78% of centres with a valve clinic had a valve multidisciplinary team meeting (MDT). 45% centres had an MDT co-ordinator and MDT outcomes were recorded on a database in 64%. COVID-19 had a major impact on valve services in 54 (95%) hospitals.

**Conclusions:**

There has been an increase in the number of valve clinics since 2015 from 21 to 68% but the penetration is still well short of the expected 100%, meaning that valve clinics only serve a small proportion of patients requiring surveillance for HVD. COVID-19 had a major impact on the care of patients with HVD in the majority of UK centres surveyed.

## Introduction

Heart valve disease (HVD) is common, affecting 11.3% of individuals over the age of 65 in the UK [1]. It is important because untreated, severe disease can lead to premature death or heart failure. It must therefore be detected early and referred at the appropriate time for intervention according to well-established guidelines [2, 3].

Care is best provided in valve clinics with multidisciplinary teams all having competencies in HVD [2, 3]. However, a survey by the British Heart Valve Society (BHVS) in 2015 [4] showed that valve clinics existed in only 60% of Heart Centres (hospitals offering cardiac surgery and transcatheter interventions) and in 11% of District General Hospitals (DGH). Since then, the need for valve clinics has been stressed by international guidelines [2, 3] and BHVS publications [5, 6]. The COVID-19 pandemic changed the way that chronic disease is managed in many different areas and presented unique challenges to patient care. The aim of this survey was two-fold; 1 to understand the national picture of heart valve disease provision, and 2, to capture how the COVID-19 pandemic impacted on the care of patients with HVD.

## Methods

### Survey creation

The BHVS Valve Clinic Survey consisted of thirty-three questions (appendix 1) covering hospital and valve service characteristics, details of multidisciplinary team (MDT) meetings, waiting times, available imaging, patient information, links to other services and the impact of COVID-19. The survey questions were designed by the authors (LD, JC) who have expertise in HVD, to assess the quality of valve clinic provision across the United Kingdom. The survey was created using commercially-available software (Qualtrics XM, Seattle, USA) using both multiple choice questions, drop down lists and free text entry boxes.

### Identification of hospitals and survey distribution

**Fig. 1:**
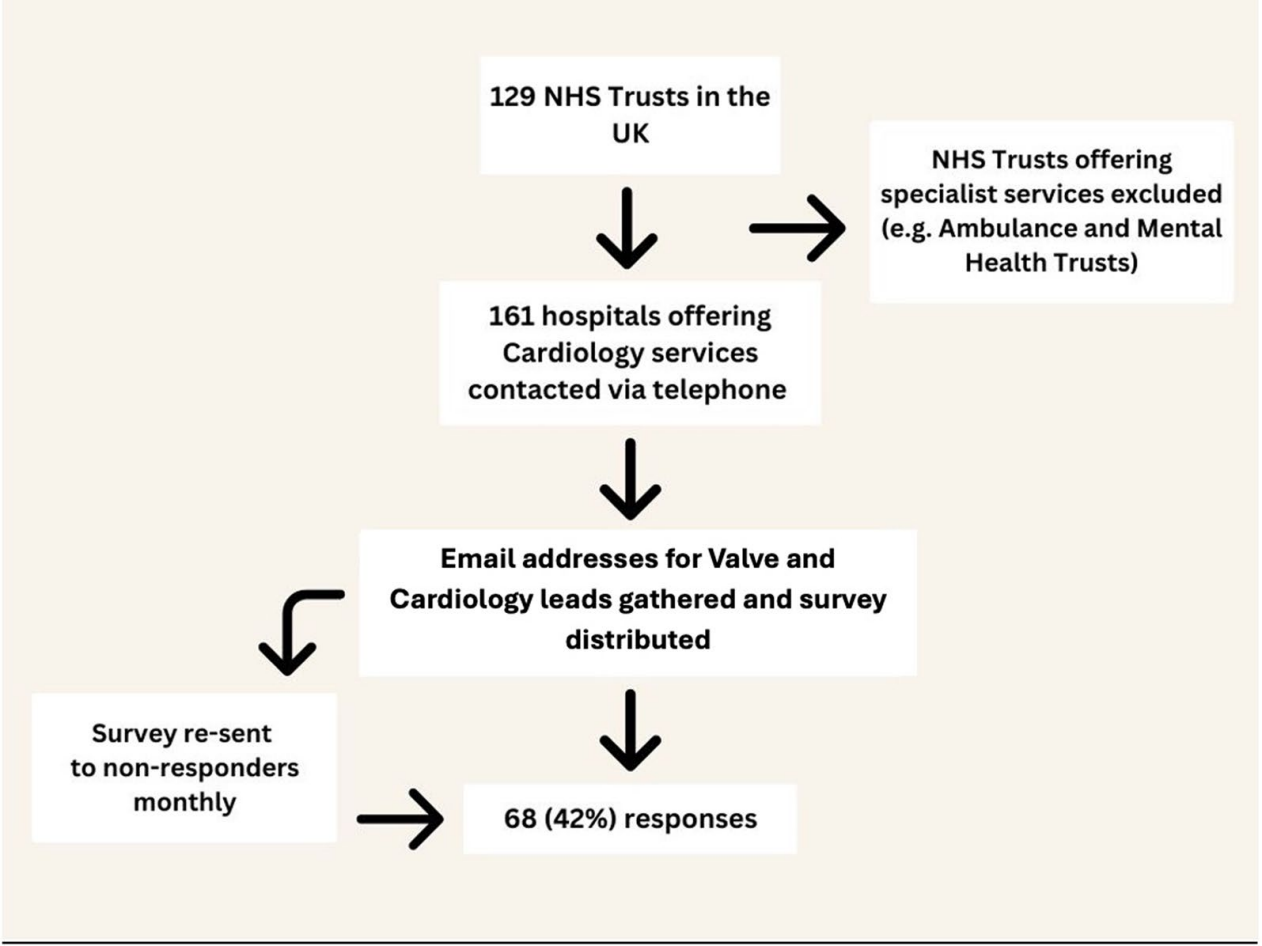
Flow diagram outlining methods for survey distribution

A list of 219 UK Trusts within the United Kingdom (England, Northern Ireland, Scotland, and Wales) was compiled using the National Health Service website (www.nhs.uk). We excluded Trusts offering only non-Cardiological specialist services (e.g. Ambulance Trusts, Mental Health Trusts). From this list we identified 125 Trusts with 161 Hospitals offering Cardiology out-patient services (Fig. 1). Appropriate contacts (usually the valve service lead if possible, or otherwise the departmental clinical lead) were identified by personal knowledge or enquiring within the respective Cardiology departments. We sent these contacts an electronic link via email and reminded non-responders on two occasions. Data were collected between September 2021 and August 2022.

Data were presented as raw values and percentages, with mean and median values and ranges where appropriate. Some questionnaires were incomplete but results of the completed sections are presented.

## Results

### Survey response rate

**Fig. 2:**
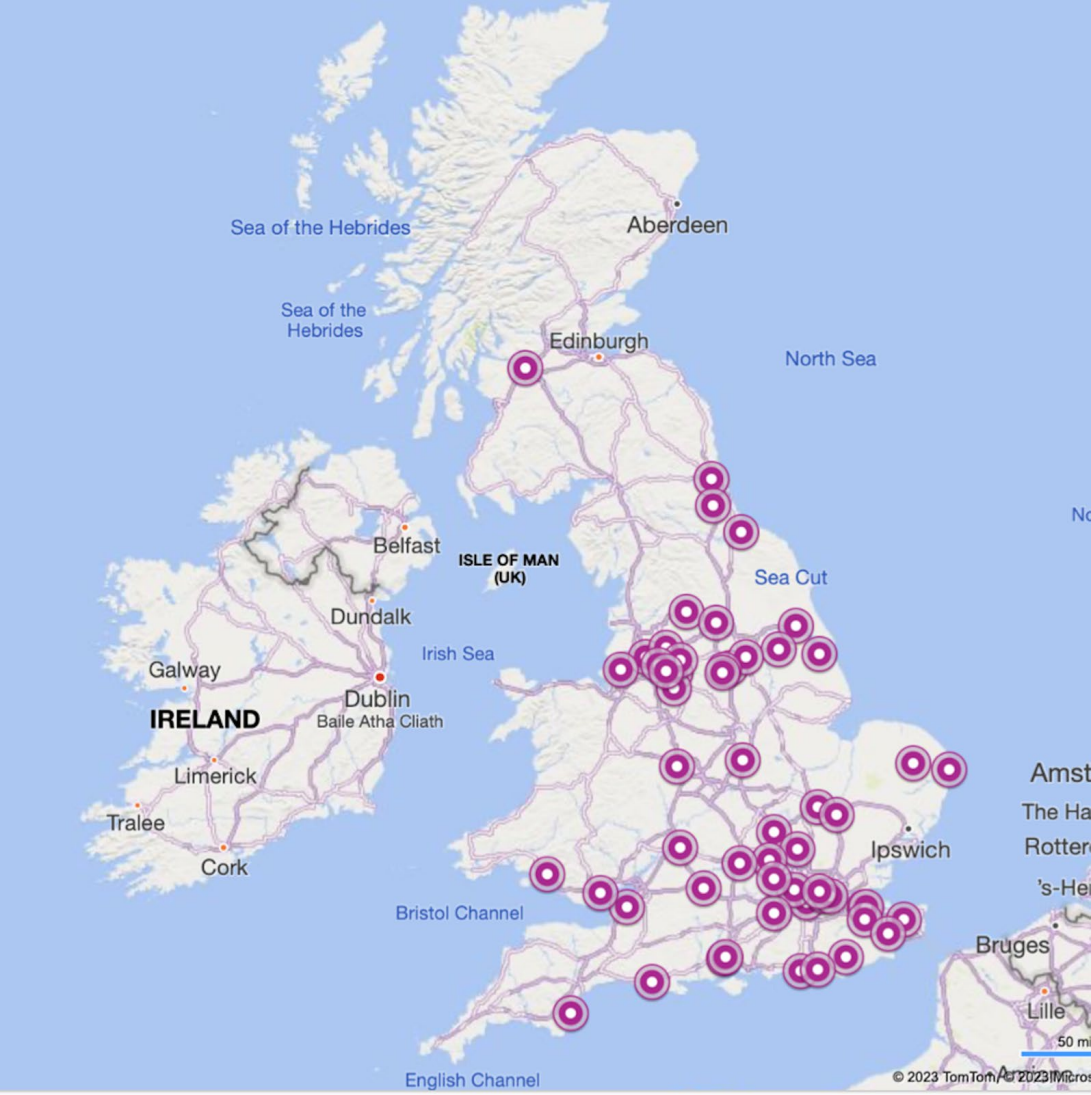
Infographic demonstrating geographical distribution of survey respondents

Of the 161 hospitals with Cardiology departments, 68 (42%) responded to the survey. Only a single reply was received from hospitals in Scotland and Wales. No replies were obtained from Northern Ireland despite involvement of local representatives (Fig. 2). There were 23 (82%) responses from 28 Heart Centres and 45 (34%) from 133 DGH.

### Valve clinics

#### General valve clinics (Fig. 3)

**Fig. 3:**
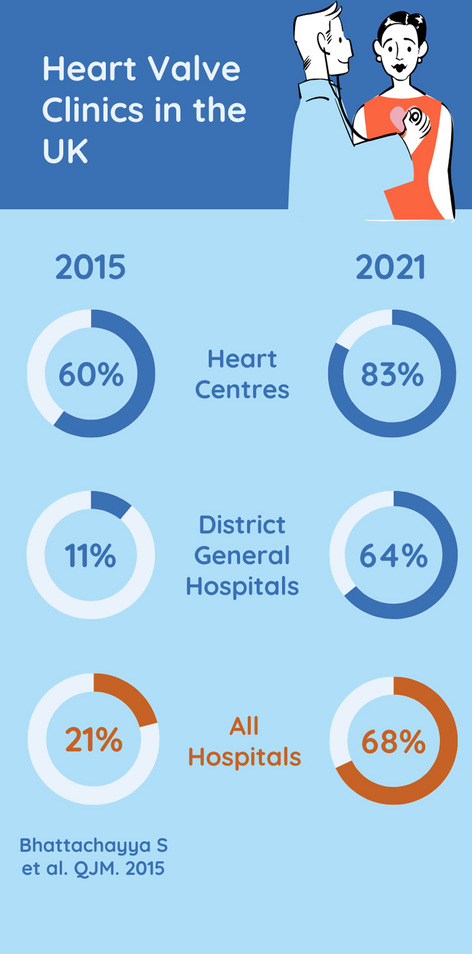
Provision of general valve clinics in 2021 compared with previous survey in 2015

There was a general valve clinic in 46 of the 68 hospitals (68%), in 19 of 23 Heart Centres (83%) and in 29 of 45 DGHs (64%). 24 of the 68 (35%) hospitals offered more than one type of valve clinic (16 Heart Centres, 8 DGH).

All 46 general valve clinics saw unoperated and post-operative valve patients, 39 (89%) followed up treated endocarditis and 36 (82%) followed up aortopathies.

#### Specialist valve clinics (Fig. 4)

**Fig. 4:**
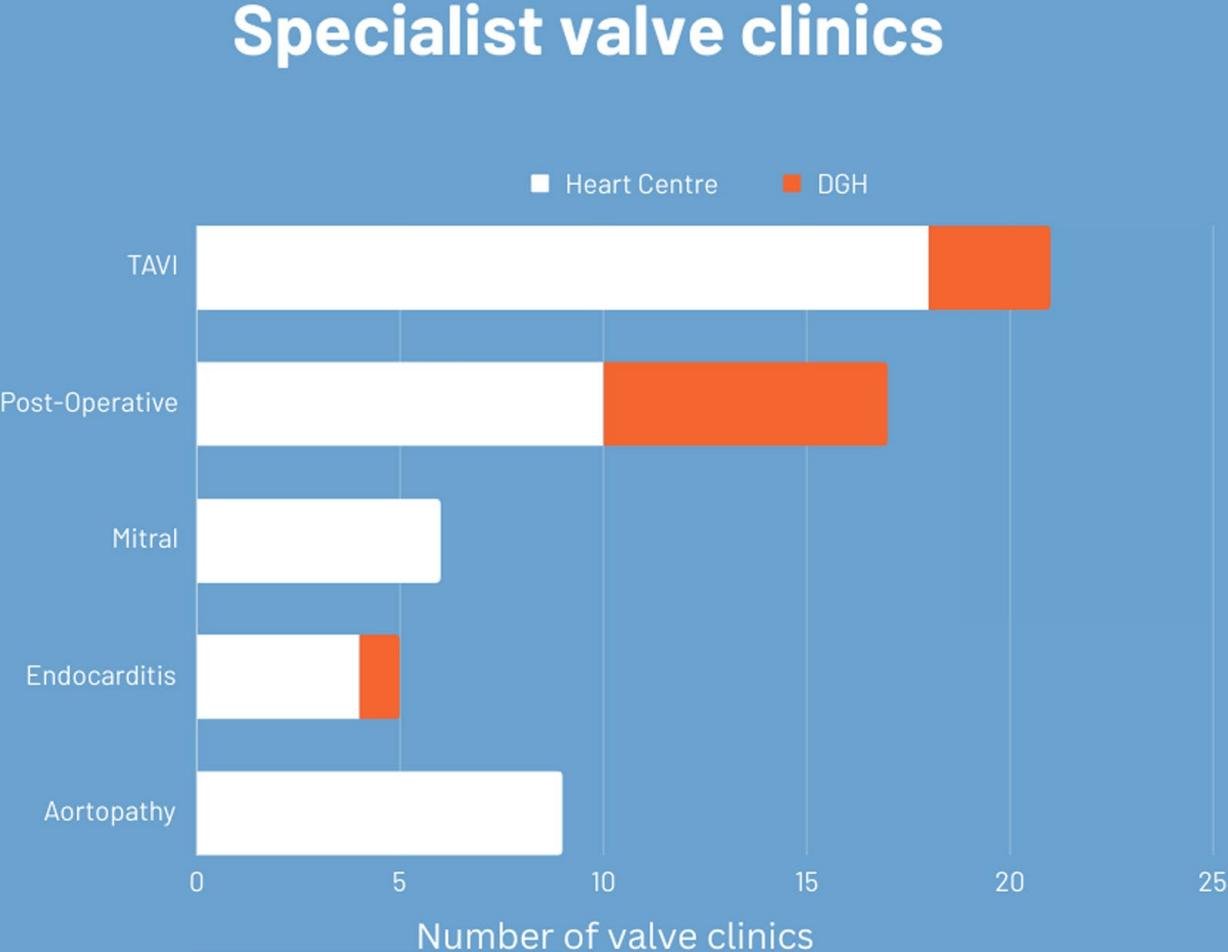
Graph demonstrating types of sub-specialist valve clinics available in Heart Centres and DGH. *DGH* District General Hospital, *TAVI* transcatheter aortic valve implantation

Specialist valve clinics were provided at some hospitals: Transcatheter Aortic Valve Implantation (TAVI) clinics in 21 (31%) (18 Heart Centres, and 3 DGH); post-operative valve surveillance in 17 (25%) (10 Heart Centres, 7 DGH); mitral specific clinics in 6 (9%) (all Heart Centres); endocarditis clinics in 5 (7%) (4 Heart Centres, 1 DGH); and aortopathy clinics in 9 (13%) (all Heart Centres). All the heart centres without a general valve clinic operated a TAVI clinic (4 out 23 (17%) Heart Centres).

#### Valve clinic activity (Fig. 5)

**Fig. 5:**
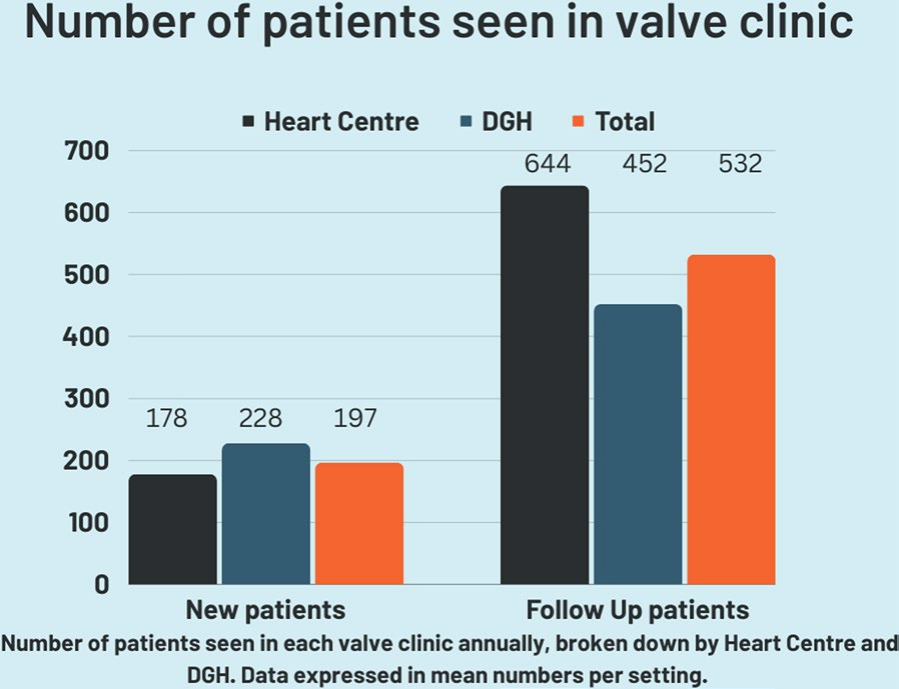
Graph demonstrating mean number of new and follow up appointments available according to DGH and tertiary centre. *DGH* District General Hospital

In absolute numbers, 3824 new patients were seen per annum across all valve clinics surveyed and 17,980 follow up patients. The mean number of new patients seen in a valve clinic in all hospitals per year was 197 (Fig. 5). At Heart Centres there was a mean 178 patients and at DGH a mean of 228. The mean number of follow-up patients each year at all hospitals with a valve clinic was 532. At Heart Centres the mean was 644 and at DGH the mean was 452.

There was a mean of 3.38 valve clinic sessions per week across all hospitals (median 2, range 0.25–20). Heart Centres had a mean 3.5 sessions (median 2, range 1–10) while DGHs had a mean 2.7 sessions (median 2, range 0.25–10).

### Specialist advice

#### Infective endocarditis

Inpatient endocarditis advice was offered in 9 of 68 hospitals (13%) by specialist teams available within working hours and a further 3 of 68 hospitals (4%) offered services both within and out-of-hours.

#### General HVD

Specialist teams for advice on general HVD were available in 9 of 68 (13%) hospitals within working hours, and 3 of these also offered out of hours advice.

#### Specialist HVD

13 of 68 (19%) hospitals had specialist teams offering TAVI patient advice within working hours, with 2 of 68 (3%) also offering out of hours specialist advice.

### Multi-professional working

Valve clinics were run by a sole clinician in (in all cases a valve consultant) in 8/46 (17%) centres. All remaining clinics were a mixture of different professionals. The multiprofessional team assessing patients in the valve clinic included an imaging cardiologist in 35/46 (76%) hospitals, an interventional cardiologist in 11/46 (24%), another cardiologist (unspecified) in 7/46 (15%), a surgeon in 4/46 (9%), specialist nurse in 13/46 (28%) and physiologist/clinical scientist in 28/46 (61%). Clinics included an imaging cardiologist and physiologist/clinical scientist together in 15/46 (33%) hospitals, an imaging cardiologist, physiologist/clinical scientist, and nurse specialist together in 5/46 (11%) or an interventional cardiologist, imaging cardiologist and nurse in 3/46 (7%). There was a wide array of different models across the UK.

### Diagnostics and patient education/resources

#### Diagnostic provision

Of the 57 centres that responded to the question around diagnostic provision, all had on-site transthoracic and transoesophageal echocardiography. Transthoracic echocardiography (TTE) was a full study in 90% and focused in 10%. TTE was always performed on the day of the clinic in 35% and mostly in 20% but only occasionally in 31% and never in 15%.

Treadmill testing was available in 54 (95%), stress echocardiography in 49 (86%), cardiovascular magnetic resonance imaging in 32 (56%), cardiac computed tomography in 47 (82%), positron emission tomography in 22 (39%) and B-type natriuretic peptide laboratory testing (BNP) in 53 (93%). BNP levels were measured routinely in patients with severe valve disease at 22 (39%) hospitals and treadmill exercise testing was performed routinely in 28 (49%).

#### Patient information/education

Patients were offered information leaflets in 35 (73%) valve clinics, website information in 18 (38%) and a helpline in 13 (27%). There was a weight loss programme in 3 (6%). Links were available to a psychologist in 5 (10%), to a dentist in 15 (31%), heart failure services in 42 (88%) and electrophysiology services in 24 (50%).

### Multidisciplinary team meetings (Fig. 6)

**Fig. 6:**
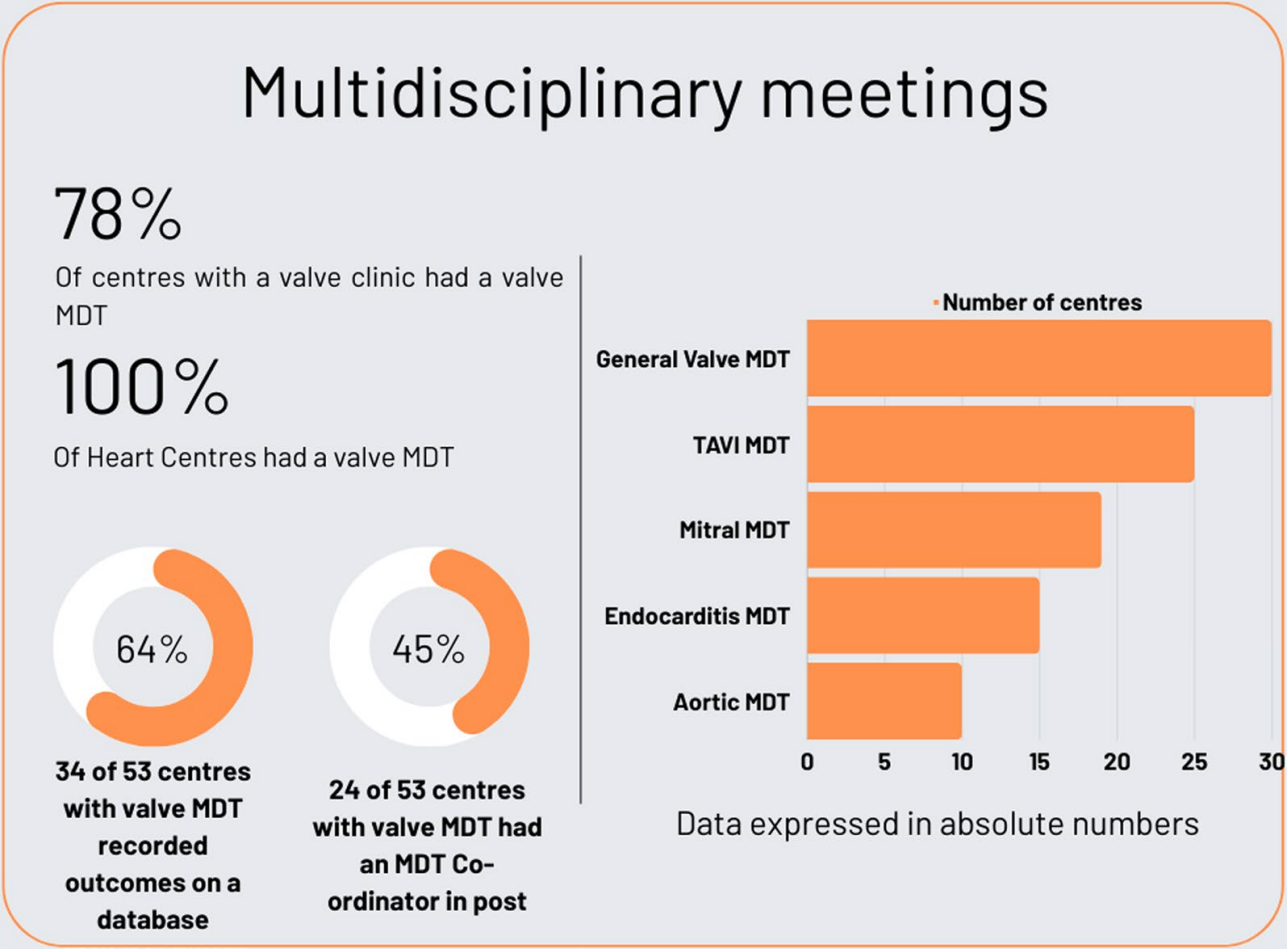
Infographic displaying main findings related to multidisciplinary team meetings. *MDT* multi-disciplinary team meeting

MDT meetings were conducted in 53 (78%) of all hospitals. MDT meetings were not conducted at 15 (22%) hospitals, all DGHs. All heart centres offered some form of MDT meeting. A general valve MDT meeting occurred in 30 (44%) hospitals, TAVI MDT meetings in 25 (37%), mitral meetings in 19 (28%), infective endocarditis meetings in 15 (22%) and aortic meetings in 10 (15%). More than one type of MDT meeting occurred at 22 (32%) hospitals. General valve MDT’s ran twice per week in once centre, weekly in 24/30 (80%) centres, bi-weekly in 3/30 (10%), 3 times per month in 1/30 (3%) and once/month in one centre.

Heart failure specialists were present at 16/30 (53%) of general valve MDT’s, 2/25 (8%) TAVI MDT’s, 4/19 (21%) of mitral MDT’s and 5/15 (33%) endocarditis MDT’s. Electrophysiologist were present at 7/30 (23%) of general valve MDT’s but not at any other type of MDT. A care of the elderly physician was present at 2/19 (11%) TAVI MDT’s but there was no representation by this staff group at any of the other MDT types.

The majority of hospitals (*n* = 41, 77%) reported that they discussed all or most valve cases undergoing surgical or transcatheter valve intervention. Cases were discussed sometimes in 10 hospitals (19%) and never in 2 hospitals (4%). The physician in charge of the case was always present in 9 (20%) MDT meetings and mostly in 26 (57%), but only sometimes in 10 (22%) and rarely in 1 (2%).

There was a designated MDT meeting manager/co-ordinator in 24 (45%) hospitals (15 Heart Centres and 9 DGH) and MDT decisions were recorded on a database in 34 (64%) (17 Heart Centres, 17 DGH). Conclusions were communicated to the patient in 34 (64%) hospitals, to the referring clinician in 42 (79%) and the GP in 34 (64%).

### Effect of COVID-19

There were 57 responses regarding the impact of the pandemic on services. COVID-19 had a major impact on valve services in 54 (95%) hospitals.

Factors classed as ‘major’ included:

- Increased out-patient appointment waiting times in 35 (61%) hospitals
- Reduction in frequency of follow-up appointments in 30 (53%)
- Shift to virtual clinics in 35 (61%)
- Delays to intervention 35 (61%) (due to a combination of catheter laboratory and bed closures and reduced ITU capacity)
- Delays to obtaining diagnostic testing in 41 (72%)
- Changes in the personnel attending MDT meetings in 10 (18%) and in case-mix in 7 (12%).
- Shift from surgical aortic valve replacement to TAVI, where technically feasible, because of reduced waiting times for TAVI and to reduce inpatient stays in 16 (57%).

## Discussion

### Heart valve clinic provision

The key finding of this survey is the major increase in the provision of valve clinics from 21% in 2015 to 68% in 2021. The largest increase was seen in DGHs, from 11% in 2015 to 64% in 2021. The increase was smaller in Heart Centres, from 60% in 2015 to 83% in 2021.

A heart valve clinic is recommended at every Heart Centre and DGH to concentrate expertise in valve disease and streamline processes including non-invasive investigation or referral for intervention [5]. Education and engagement of patients is also an important role of a valve clinic so that the process of consent occurs potentially over many visits and consists of an individualised dialogue rather than a generic didactic statement of risks and benefits [7]. Most hospitals offered some sort of patient information usually as leaflets or web-resources. This needs to be extended to include other material to suit all educational levels and this is a project of the BHVS.

Valve clinics lead to earlier detection of symptoms [8] and are liked by patients [9, 10]. The improvement seen between 2015 and 2021 is therefore welcome but not enough. There were four Heart Centres with a TAVI clinic alone without offering a general valve clinic and this is not sufficient. A valve clinic is defined in all guidelines as a general valve clinic seeing all patients with native and operated valve disease. In our view, the minimum offering at every centre should be a general valve clinic, run by a valve specialist with expertise in all aspects of care of patients with HVD. On the foundation of these clinics, other clinics providing specialist services can be developed, including specialist offerings such as TAVI clinics, which are able to offer prompt assessment and treatment on an individualised basis. BHVS has published a guide to setting up a heart valve clinic [6].

### Valve clinic activity

The valve clinics at all hospitals saw a mean 200 new and 500 follow-up patients each year. DGHs saw more new patients each year, mean 228, compared with Heart Centres, mean 178. By contrast Tertiary Centres saw more follow-up cases each year, mean 644, compared with DGHs, mean 452. The larger number of follow-up cases at Tertiary Centres may reflect surveillance after intervention. The differences in new cases is harder to explain since the majority of Heart Centres also serve their community as a secondary hospital like any DGH.

The patients in these valve clinics represent only the ‘tip of the iceberg’ of the burden of heart valve disease in the UK. Over 300,000 patient in the UK are estimated to have severe aortic stenosis alone [11]. Absolute numbers of patients across the UK seen in a valve clinic represented just a fraction of this figure; with only around 4000 new patients and 18,000 follow up patients being seen in HVD clinics in our survey. Every patient across the UK with significant heart valve disease should be monitored in a heart valve clinic, and the numbers seen in the clinics in this survey fall far short of community prevalence. Detection in the community needs to be greatly improved.

### Diagnostic provision at heart valve clinics

Processes within HVD remain suboptimal. The most obvious problem is that TTE were performed always or mostly on the same day as the clinical assessment in only in 55% of clinics compared with 82% in 2015, a facility which is very much preferred by patients and has the opportunity to reduce health inequalities by reducing frequency of hospital visits (and therefore costs), in addition to potentially reducing ‘do not attend’ rates. Despite the logistical challenges of providing this service as part of a valve clinic, services should focus efforts to maintain or adapt this important service structure as per the ‘NHS England Getting it right first time out-patient guidance’ [12]. Transthoracic echocardiography was performed as a full study in most centres. Uptake of focussed echocardiography to assess patients under valve surveillance will depend on local expertise and resource, and its utility is debated within the echocardiographic community which may account for its limited uptake at present. Access to other ‘on-site’ diagnostics was good, with most valve clinics having access to stress echocardiography, cardiac computerised tomography, and BNP. It is clear that patients with severe disease should be heavily scrutinised to ensure that they are not displaying subtle signs of deterioration, and therefore disappointing that only 39% of patients with severe heart valve disease had regular evaluation of serum BNP levels and only 49% had exercise testing. However, these figures are better than the EuroValve survey [13] in which only 10.5% had exercise testing. The need for exercise testing to unmask sub-clinical symptoms is all-the-more important in the current period of COVID-19 recovery, where waiting lists for valve intervention in the UK are long and patients are more frequently deconditioned [14].

### MDT meetings

Valve MDT’s of some iteration were available at 78% of Hospitals surveyed, although the type of MDT offered varied according to centre. Although methodology differed with a previous survey by the BHVS in 2014, it is likely that valve MDT offerings have increased over the years, with only 18% of centres offering a valve MDT in 2014 [15].

The British Cardiovascular Society and BHVS have published guidance on MDT meetings [5, 16]. Requirements were being met in some centres including the existence of an MDT co-ordinator, the recording of conclusions on a database and communication with patient, GP, and referring clinician. However, there are still hospitals not meeting this standard and concerningly, MDTs are still taking place without the clinician in charge of the case being present. This is a major failing since the individual circumstances and the opinion of the patient are vital to making a valid decision and should only be permissive in exceptional rather than routine circumstances. 77% of MDT meetings discussed all or most patients being considered for surgical or transcatheter valvular intervention. This is encouraging and represents a welcome shift in clinical practice. Only 64% of MDTs communicated the result of the discussions with the patient. In our view all patients should receive communication of the outcome of the MDT in a manner they can understand at what is often an extremely anxious time for them.

### Endocarditis

Despite contemporary infective endocarditis guidelines suggesting that all patients with endocarditis are discussed with the ‘endocarditis team’, few centres offered specialist in-patient endocarditis advice (13%) or access to an endocarditis MDT (22%). This is a concerning finding given the class I recommendations for this in the latest ESC guidelines, and demonstrates that systematic change is required across the UK to improve access to these services which can often make an important difference to patient care [17]. Pockets of good practice were seen, however, and can be expanded across the UK with the advocacy of groups such as the BHVS.

### Impact of COVID-19

The COVID-19 pandemic had a serious impact on the care of patients with HVD. It reduced the frequency of follow up, and access to diagnostics and introduced delays to intervention in the majority of hospitals. Given that severe symptomatic aortic stenosis has a prognosis worse than most metastatic cancers, this is likely to have had an adverse effect on patient survival and morbidity [14, 18]. A shift towards percutaneous valve treatment as a direct result of the pandemic was reported in almost a third of Heart Centres. Although this may have been influenced by a shifting evidence base, changing routine practice purely in response to service pressure rather than clinical factors, should be cautioned against.

### Limitations

Simple quantitative data on service provision was recorded but we did not capture data on the ‘quality’ of the valve services such as appropriateness of tests or adherence to published national and international guidelines.

Although efforts were made to capture all cardiology departments across the UK, some hospitals may have been omitted. NICOR [19] lists 168 hospitals offering pacing as an example of another key cardiac service. We approached 161 of these hospitals so might have missed 7 [19]. The response rate of 42% was reasonable for a survey of this design. It is possible that hospitals offering a more comprehensive valve service were more likely to respond than those without. The response rate from Scotland, Wales and Northern Ireland was very poor and therefore the results are mostly reflective of English, not UK practice. The response rate for English hospitals was 54%. Finally, some of the questions required data estimates (i.e. number of new-patient and follow up appointments), an as such subject to potential inaccuracy.

## Conclusion

There has been an increase in the number of valve clinics since 2015 but the penetration is still well short of the expected 100%, meaning that ‘gold-standard’ valve clinics only serve a very small proportion of patients requiring surveillance for HVD. Guideline-recommended surveillance including routine BNP and exercise testing in patients with severe HVD is only performed in less than half of heart valve clinics. National guidelines mean that MDT provision is good across the UK and the majority of patients undergoing valve intervention are discussed in this setting. The COVID-19 pandemic has had a detrimental effect on the provision of services for patients with HVD in general, although the rapid development of the ‘virtual’ MDT meeting has meant that a ‘hub and spoke’ model in HVD care has developed effortlessly.

## Data Availability

No datasets were generated or analysed during the current study.

## Appendix 1. Table of survey questions

**Table:**

*PAGE 1*
What is the name of your hospital? - *(Text Entry)*
Is there on-site cardiac surgery? - Yes - No
How many whole-time equivalent consultant cardiologists work in your department? - *(Text/Numerical entry)*
Does your department have a specialist heart valve clinic? (Please select all that apply) - Yes—General Valve Clinic (accepting many different pathologies) - Yes—TAVI - Yes—Specialist Mitral Valve Clinic - Yes—Post-operative surveillance - Yes—Endocarditis Clinic - Yes—Aortopathy Clinic - No
Which types of patients are seen in your general valve clinics? (Please select all that apply) - Native Unoperated - Post TAVI - Post Valve Replacement - Post Valve Repair - Post Endocarditis - Aortic Pathology
How many general valve clinics are there per week? (approximately) - *(Text/Numerical entry)*
Which health care professionals participate in valve clinics? (Please select all that apply) - Cardiologist—Imaging - Cardiologist—Interventional - Cardiologist—Other - Cardiothoracic Surgeon - Specialist Nurse - Physiologist/Scientist
Approximately, how many new cases are seen per year? - Don’t know - Please specify estimated number *(Text/Numerical entry)*
Approximately, how many follow up cases are seen per year? - Don’t know - Please specify estimated number *(text/numerical entry)*
Is there provision of specialist on call services for patients with valve disease? - Yes—Endocarditis (Options for day provision and/or night provision) - Yes—All valve patients (Options for day provision and/or night provision) - Yes—TAVI cases (Options for day provision and/or night provision) - No—General Cardiology cover only (Options for day provision and/or night provision) - Other—please enter text below *(text entry)*
Is there provision of specialist services offering inpatient opinions for patients with valve diseases? (→ If any yes box is checked, please specify if these are ad-hoc or timetabled) - No—General Cardiology cover only - Yes—Endocarditis *(text entry)* - Yes—All Valve patients *(text entry)* - Yes—TAVI cases *(text entry)*
*PAGE 2*
Do you have valve MDT meetings? (Please select all that apply) - Yes—General Valve MDT - Yes—TAVI / Aortic Valve meeting - Yes—Mitral Valve meeting - Yes—Endocarditis Clinic - Yes—Aortopathy Clinic - No
Are all valve patients undergoing valve intervention (surgery or transcatheter procedure) discussed at MDT? - Yes—Always - Yes—Mostly - Sometimes - Occasionally - Rarely - No - N/A
Which clinicians attend the MDT meetings? - Valve Cardiologists o ± General Valve MDT o ± TAVI MDT o ± Mitral Valve MDT o ± Endocarditis MDT o ± Aortopathy MDT - Interventional Cardiologists o ± General Valve MDT o ± TAVI MDT o ± Mitral Valve MDT o ± Endocarditis MDT o ± Aortopathy MDT - Physiologist / Scientists / Sonographer o ± General Valve MDT o ± TAVI MDT o ± Mitral Valve MDT o ± Endocarditis MDT o ± Aortopathy MDT - Surgeons o ± General Valve MDT o ± TAVI MDT o ± Mitral Valve MDT o ± Endocarditis MDT o ± Aortopathy MDT - Heart Failure Physician / Specialists o ± General Valve MDT o ± TAVI MDT o ± Mitral Valve MDT o ± Endocarditis MDT o ± Aortopathy MDT - Care of the elderly Physician / Specialists o ± General Valve MDT o ± TAVI MDT o ± Mitral Valve MDT o ± Endocarditis MDT o ± Aortopathy MDT - Electrophysiologists o ± General Valve MDT o ± TAVI MDT o ± Mitral Valve MDT o ± Endocarditis MDT o ± Aortopathy MDT - Specialist Nurse o ± General Valve MDT o ± TAVI MDT o ± Mitral Valve MDT o ± Endocarditis MDT o ± Aortopathy MDT - Other, please specify *(text entry)* o ± General Valve MDT o ± TAVI MDT o ± Mitral Valve MDT o ± Endocarditis MDT o ± Aortopathy MDT
*PAGE 3*
Approximately, how many MDT meetings are there per month? (0–10 scale: Please select all that apply and drag bar to estimated number) - TAVI MDT - Mitral Valve MDT - General Valve MDT - Endocarditis MDT - Aortopathy MDT
Is there a MDT manager/co-ordinator? - No - Yes—please specify the profession of the manager *(text entry)*
Is the clinician who assesses the patient present? - Always - Most of the time - Sometimes - Rarely - Never
Are clinical decisions recorded on a database? - Yes - No
Who are decisions communicated to? - Patient - Referring practitioner - General Practitioner - Other, please specify *(text entry)*
*PAGE 4*
What is the average waiting time for patients who require urgent outpatient surgery? (If not known please mark as N/A) - *(text/numerical entry)*
What is the average waiting time for patients who require a TAVI? (If not known please mark as N/A) - *(text/numerical entry)*
What is the average waiting time for patients who require a transcatheter mitral procedure? (If not known please mark as N/A) - *(text/numerical entry)*
Which of the following imaging/investigative modalities are offered on-site at your hospital? (Please select all that apply) - Transthoracic echocardiography - Transoesophageal echocardiography - Stress echocardiography - Cardiac magnetic resonance imaging - Cardiac computed tomography - Positron emission tomography - B-type peptide or other biomarker - Treadmill testing - Other, please specify *(text entry)*
Do you routinely check BNP in patients with severe asymptomatic valve disease? - Yes - No
Do you routinely offer exercise testing to patients with severe asymptomatic valve disease? - Yes - No
Is a Transthoracic echo provided on the same day as the assessment in the valve clinic (one stop)? - Yes—Always - Yes—Mostly - Yes—Occasionally - Never
(if YES to above question) Are these echocardiograms? - Full TTE studies - Focussed studies
Which of the following additional patient services do you offer? (Please select all that apply) - Patient information booklets - Patient website information - Patient helpline (telephone or email) - Link to weight loss programmes - Links to psychologist - Links to dental department - Links to heart failure service - Links to electrophysiology service - None of the above - Other, please specify *(text entry)*
*PAGE 5*
Have your heart valve and/or endocarditis services been affected by COVID-19? - Yes - No
How has COVID-19 impacted your services? (Please select all that apply) - The frequency of clinic delivery has been affected - The clinic format delivery has been altered (from face-to-face consultations to virtual) - The composition of clinicians at the MDT meetings has been changed - Access to patients/imaging/investigations available have been affected/delayed - Treatment/Surgery/TAVI procedures have been affected - The number and/or types of valvular heart disease cases discussed and managed have been affected - The lack of critical care beds has led to increased waiting times - The ward closures due to COVID-19 outbreaks had resulted in longer delays for valve intervention - The catheter laboratory closures have led to increased waiting times - The delays to diagnostics have resulted in increased waiting times - There has been a change in practice for more referrals to transcatheter therapy (TAVI or Mitraclip) from traditional surgical techniques - Further comments, please enter below (*free text box)*
Have new or updated guidelines been provided with respect to managing patients during the current pandemic? - Yes - No
Would you be happy to be contacted by the BHVS for further surveys and information from the society? - Yes - No
Please enter your email address below - *(Free text box)*
